# 31.3 CLINICAL UTILITY OF MRI SCANNING IN FIRST EPISODE PSYCHOSIS

**DOI:** 10.1093/schbul/sby014.129

**Published:** 2018-04-01

**Authors:** Paola Dazzan, AAT Simone Reinders, Vasiliki Shatzi, Francesco Carletti, Celso Arango, Wolfgang Fleischhacker, Silvana Galderisi, Armida Mucci, Birte Glenthoj, Alice Egerton, Gareth Barker, Stefan Leucht, Andreas Meyer-Lindenberg, René S Kahn, Dan Rujescu, Iris Sommer, Inge Winter, Philip McGuire

**Affiliations:** 1 Institute of Psychiatry, Psychology and Neuroscience, King’s College London; 2 King’s College London; 3 Hospital General Universitario Gregorio Marañon; 4 Medical University Innsbruck Biological Psychiatry Division; 5 University of Naples SUN; 6 University of Copenhagen Center for Neuropsychiatric Schizophrenia Research; 7 Technical University of Munich; 8 Central Institute of Mental Health, Mannheim; 9 University Medical Center Utrecht; 10 University of Halle; 11 UMC Groningen

## Abstract

**Background:**

The response to antipsychotic treatment in patients with psychosis is difficult to predict on the basis of the patient’s clinical features. As a result, patients are generally treated in a similar way, even though their response can vary dramatically.

Recent neuroimaging studies suggest that the pattern of brain abnormalities in patients with psychosis may vary in relation to treatment response. However, in many of these studies, patients had already been treated, and it was unclear if this had contributed to the findings.

**Methods:**

In Optimise we obtained a structural Magnetic Resonance Imaging data from n=203 minimally treated patients at their first presentation for a psychotic episode. All patients then started treatment with standard doses of amisulpride. After 4 weeks, 56% were in symptomatic remission.

**Results:**

We identified brain neoplasms in 3 patients, but the most common radiological findings were non-specific white matter T2-weighted hyperintensities (n=48); cavum septi pelludici (n=34); and arachnoid cysts (n=9).

Cortical thickness, surface area, and gyrification were measured using Freesurfer (). Preliminary analyses applying machine learning to these measures at baseline indicated that symptomatic remission at 4 weeks could be predicted with an accuracy of 64%.

**Discussion:**

These findings suggest that radiological assessment can identify abnormalties that require an alternative to conventional treatment in a minority of patients. In most patients with psychosis, neuroimaging abnormalities may be better detected using statistical approaches, and these have greater potential for the stratification of patients according to future antipsychotic response.

